# Care pathways of individuals with tuberculosis before and during the COVID-19 pandemic in Bandung, Indonesia

**DOI:** 10.1371/journal.pgph.0002251

**Published:** 2024-01-02

**Authors:** Lavanya Huria, Bony Wiem Lestari, Eka Saptiningrum, Auliya Ramanda Fikri, Charity Oga-Omenka, Mohammad Abdullah Heel Kafi, Benjamin Daniels, Nathaly Aguilera Vasquez, Angelina Sassi, Jishnu Das, Ira Dewi Jani, Madhukar Pai, Bachti Alisjahbana

**Affiliations:** 1 Department of Epidemiology, Biostatistics and Occupational Health, McGill University, Montreal, Canada; 2 McGill International TB Centre, Montreal, Canada; 3 Tuberculosis Working Group, Faculty of Medicine, Universitas Padjadjaran, Bandung, Indonesia; 4 Department of Public Health, Faculty of Medicine, Universitas Padjadjaran, Bandung, Indonesia; 5 School of Public Health Sciences, University of Waterloo, Waterloo, Canada; 6 McCourt School of Public Policy, Georgetown University, Washington, DC, United States of America; 7 School of Human Nutrition, McGill University, Ste. Anne-de-Bellevue, Quebec, Canada; 8 Department of Internal Medicine, Faculty of Medicine, Universitas Padjadjaran, Hasan Sadikin General Hospital, Bandung, Indonesia; Johns Hopkins Center for Health Security: Johns Hopkins University Center for Health Security, UNITED STATES

## Abstract

The COVID-19 pandemic is thought to have undone years’ worth of progress in the fight against tuberculosis (TB). For instance, in Indonesia, a high TB burden country, TB case notifications decreased by 14% and treatment coverage decreased by 47% during COVID-19. We sought to better understand the impact of COVID-19 on TB case detection using two cross-sectional surveys conducted before (2018) and after the onset of the pandemic (2021). These surveys allowed us to quantify the delays that individuals with TB who eventually received treatment at private providers faced while trying to access care for their illness, their journey to obtain a diagnosis, the encounters individuals had with healthcare providers before a TB diagnosis, and the factors associated with patient delay and the total number of provider encounters. We found some worsening of care seeking pathways on multiple dimensions. Median patient delay increased from 28 days (IQR: 10, 31) to 32 days (IQR: 14, 90) and the median number of encounters increased from 5 (IQR: 4, 8) to 7 (IQR: 5, 10), but doctor and treatment delays remained relatively unchanged. Employed individuals experienced shorter delays compared to unemployed individuals (adjusted medians: -20.13, CI -39.14, -1.12) while individuals whose initial consult was in the private hospitals experienced less encounters compared to those visiting public providers, private primary care providers, and informal providers (-4.29 encounters, CI -6.76, -1.81). Patients who visited the healthcare providers >6 times experienced longer total delay compared to those with less than 6 visits (adjusted medians: 59.40, 95% CI: 35.04, 83.77). Our findings suggest the need to ramp up awareness programs to reduce patient delay and strengthen private provide engagement in the country, particularly in the primary care sector.

## Introduction

Indonesia has the world’s third highest tuberculosis (TB) burden. While estimated incidence was 969 000 in 2021, an estimated 536 423 people (55.3%) [1] people remain undiagnosed or diagnosed but not notified to the National TB Program (NTP), otherwise referred to as, ‘missing people with TB.’ Locating the missing people with TB has remained a challenge for decades [2], and has been deemed the single biggest barrier to TB control in Indonesia [3]. The COVID-19 pandemic has only widened this gap, and Indonesia saw the second biggest drop in TB notifications in the world; between 25–30% fewer cases were notified in the first six months of 2020 as compared to 2019 [4]. This was repeated once again in 2021, and Indonesia contributed to 18% of the reduction in global TB case notifications [1].

The Indonesia High-Frequency Monitoring of COVID-19 Impact Survey, conducted from May-August 2020, found that 11% of households that needed medical treatment for any reason were unable to access it, citing closures of facilities, lack of money, and unwillingness to seek healthcare due to the COVID-19 pandemic as the main obstacles. Furthermore, 17% of households that were on TB treatment were unable to access that treatment in August 2020 [5]. The Ministry of Health, Republic of Indonesia reported that TB treatment coverage decreased from around 60% in 2019 [4] to 47% in 2020 [6–8] and 45% in 2021 [1]. The partial recovery for case notifications that was seen in 2021 was overshadowed by the increase in incidence [1]. These declines in case notifications and treatment coverage are consistent with the COVID-19 pandemic seriously disrupting TB care. However, the full picture of how COVID-19 affected TB notifications is difficult to obtain from official data alone since 74% of initial care-seeking for TB takes place in the private healthcare sector, where underreporting of TB is a serious concern [3]. Only 9% of cases that originated from the private sector were reported to the NTP in 2015 [3], and in 2017, this figure was only marginally higher at 13% [3, 9, 10].

An alternate approach to understanding the impact of COVID-19 is to use surveys of individuals with TB to obtain detailed information on healthcare seeking behaviours. These surveys, known as patient pathway analyses (PPA), summarise service delivery by measuring the quantity, sector, and level of providers encountered by the patient on their journey towards diagnosis and treatment; at an aggregate level, they provide system-level overview of delays in diagnosis and treatment [2]. Two previous (pre-pandemic) PPAs from Indonesia discovered that as individuals with TB transition from one healthcare provider to the next and have multiple encounters, the delays to diagnosis and treatment initiation increase, resulting in worse health outcomes, increased transmission to closed contacts, and higher direct and indirect costs [11–17]. However, among others, limitations of conducting patient pathway analyses in the conventional manner are that they cannot measure the duration of delays that individuals face while navigating the journey towards their recovery, they simplify the individual’s journey when in fact, the pathway is possibly more complex, and they cannot capture the factors associated with higher delays or complex pathways without cross-referencing individual-level data. Moreover, the COVID-19 pandemic has added a layer of complexity, as the effects of the restrictions and lockdown protocols on delays and care pathways have not yet been fully studied.

This PPA is one of the few such studies conducted during the pandemic phase for TB. In this study, we aimed to quantify the delays that individuals with TB faced while trying to access care for their illness, before and during the COVID-19 pandemic, their journey through TB diagnosis and treatment, the encounters individuals had with healthcare providers for a TB diagnosis, and examine the factors associated with patient delay and the number of encounters.

## Methods

### Study setting, population, and COVID-19 wave

We performed two cross-sectional surveys in Bandung, West Java province, Indonesia in 2018 and then again in 2020–22. The first survey in 2018, was part of the INSTEP study (Investigation of services delivered for TB by external care system, especially the private sector) in Bandung, over a 2-year period [18]. The second survey in 2020–2022 was part of the COVID-19 effect on Tuberculosis (COVET) project, which aimed to examine the disruptions caused by the COVID-19 pandemic on TB services in private healthcare in 3 countries–India, Indonesia and Nigeria [19]. Over this time, TB cases notified to the NTP nationwide declined to 443 235 (in 2021) from 570 289 (2018).

The setting of our surveys is Bandung, the third largest city in the country, located southeast of Jakarta with a population of 2,606,850, which is 2.71% higher in 2021 compared to 2018. In Bandung, TB services are supplied through both public and private sector providers. In 2021, there are 80 community health centres (CHCs), 358 clinics, 22 secondary level hospitals, 15 tertiary level hospitals. Only 6 CHCs, 5 hospitals, and 1 public laboratory have Xpert/MTB-RIF as a molecular test for TB diagnosis, which is the diagnostic test primarily recommended by the National TB Program (NTP) for diagnosing TB. It is unclear how many private clinics and practitioners have diagnostic facilities.

The individuals in both datasets are adults (>18 years of age) living in Bandung who have been previously diagnosed with TB and were on TB treatment at the time of recruitment. As individuals were from several healthcare providers across Bandung, no clustering at specific healthcare facilities was suspected, and to limit recall bias, all individuals were surveyed within 6 months of their treatment initiation.

### Sampling, enrolment, and data collection

**Fig 1:**
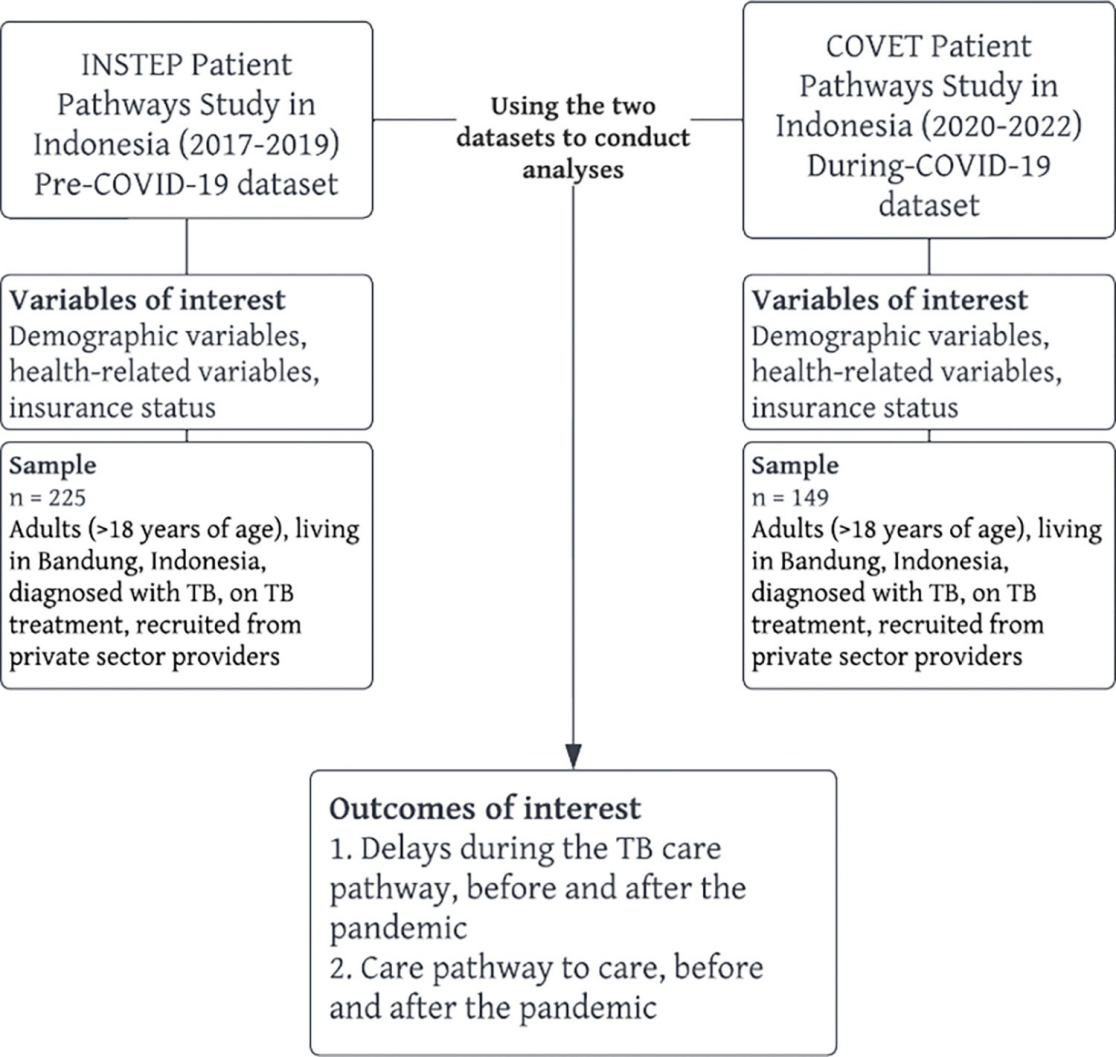
Flowchart depicting pre- and during-COVID-19 datasets.

A more detailed description of the sampling procedure for the INSTEP project, the pre-COVID-19 dataset, is given in the article by Lestari et al [14]. Sampling for the COVET project was designed to closely replicate the INSTEP project. The researchers used hierarchical sampling, starting from sub-districts of Bandung. Bandung contains 151 sub-districts, from which 30 were randomly selected using probability proportional to size sampling for both INSTEP and COVET. Four private hospitals and 59 private practitioners were selected to for the COVET study, based on high TB case density at the clinic/hospital, and providers’ willingness to participate in the study. Enrolment into the study depended on the individual being a patient in one of the private hospitals or private practitioners. Ultimately, the study includes 225 respondents from the INSTEP dataset (80 recruited from private hospitals and 145 from private clinics). In the COVET project, sample sizes were pre-determined by funding and time constraints. Consenting patients were enrolled from 7^th^ July 2021 until 28^th^ February 2022, until the desired sample size was met. Some public hospitals and community health centres were also sampled for a separate study. However, this study only analyses data from respondents recruited from private sector facilities. The COVET study conducted 61 (40.9%) online interviews, while all 225 (100%) interviews in INSTEP were conducted offline. Information regarding the sample from the two datasets is described further in **Fig 1.** Individuals who had previous history of being on TB treatment, extrapulmonary TB, and those residing outside the study site (Bandung) were excluded. The during-COVID-19 sample size was fixed at 149 participants as recruitment numbers were decided across 3-countries in the primary COVET study and limited by the grant budget. With the two fixed sample sizes, we had a power of 93.8% to detect our main outcome—difference in patient delay (in days) due to the pandemic (Cohen’s *d*, estimated at 0.37), at the 5% alpha level. For the secondary outcome of number of encounters before diagnosis, we had a power of 99.7%, at the 5% level, to detect *d* of 0.5. Verbal consent was obtained at the time of interview by the interviewer.

The questionnaire aimed to collect information on socio-demographic characteristics, clinical symptoms of their TB disease, healthcare seeking pathway, and pre-diagnostic cost information. The tool was conducted in Bahasa, the official and national language of Indonesia. Informed consent was obtained at the start of the interview. Most interviews were conducted in-person, in the recruitment healthcare facility or at the participant’s house, as per the respondent’s request. However, during periods of high COVID-19 burden, the interviews were carried out through WiFi voice calls. Participants received monetary compensation in the form of cash or bank transfer for transportation or internet credit costs.

### COVID-19 and recruitment

Respondents were recruited between July 2021 and February 2022. Recruitment was slower during periods of high COVID-19 burden, and the most active between the months of September 2021 and January 2022 as depicted in **S1 Fig**. The interviewers faced restrictions when COVID-19 cases peaked, and therefore had to pause in-person interviews and shift to online phone interviews. Data collection was not significantly affected, and the interviews took a similar amount of time to conduct. COVID-19 cases displayed a cyclical pattern of rise and fall every couple of months, with the largest rise in cases at the end of recruitment, and the second highest peak in July 2021. Our sample represents care-seeking throughout the year, from January 2021 till the end of February 2022; and individuals were also being diagnosed during these times of heightened burden and recovery. Therefore, the COVID-19 variable was treated as a binary variable, either present or not-present.

### Outcomes of interests and definitions

**Fig 2:**
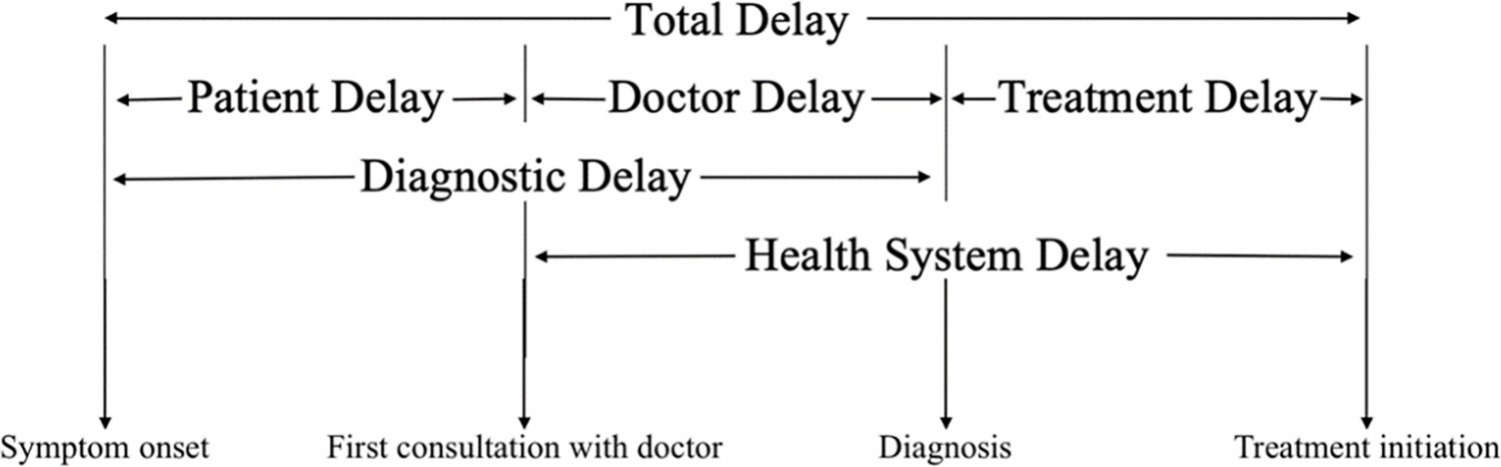
Definition of delays in care-seeking, diagnosis and treatment of pulmonary tuberculosis, figure recreated for clarity, adapted from World Health Organisation [20].

The main outcomes of interest were delays and number of encounters. The various types of delays are presented in the **Fig 2**. *Patient delay* is defined as the number of days between the onset of symptoms and first consultation with healthcare provider, *doctor delay* is defined as the number of days from that first consultation until the participant was given a diagnosis for their TB, and *treatment delay* is the number of days between the date of diagnosis and date of TB treatment initiation. *Encounter* is defined as a visit to a healthcare provider (of any level, from community pharmacist to specialist at a tertiary care hospital). The different kinds of healthcare providers that individuals visited during their care pathway are private clinics, private hospitals, public hospitals, community pharmacies, CHCs (*Puskesmas*) traditional healers and medicine shops, and public and private laboratories. Community pharmacies are defined as stand-alone shops, with registered pharmacists present, and traditional healers and medicine shops are informal healthcare providers, more locally known.

### Statistical analysis

All data cleaning, visualisation, and statistical analysis was performed on R [21]. Numerical variables were described by median and interquartile range (IQR) for age and average household monthly income, and categorical variables were described by proportion (percentage).

Univariable regression was used to first investigate factors associated with patient, diagnostic, and treatment delay and numbers of encounters until diagnosis, and then a multivariate model was fitted to control for confounders and investigate the effect of the COVID-19 pandemic on the outcomes of interest. Age, gender, education level, employment status, average household income, insurance status, comorbidities, minutes to nearest CHC, cough duration, and having a fever were included as risk factors for delays. Additionally, we inquired about all the *encounters* the individual had with various healthcare providers leading up to their diagnosis. Specifically, the purpose of the visit, the type of provider, and the provider’s sector.

Due to the outliers in the data and the skewed but unimodal underlying distribution of the continuous outcome variables, quantile regression (*tau* = 0.5) was utilised to examine the association between the outcome of interest (patient delay, doctor delay, treatment delay, number of encounters) and their associated factors. Univariable regression was used to first investigate factors associated with patient delay and numbers of encounters until diagnosis, and then a multivariate model was fitted to control for confounders and investigate the effect of the COVID-19 pandemic on the outcomes of interest. The associated factors for the patient delay model were chosen based on the previous manuscript by Lestari et al [14] and after reviewing literature regarding the most common factors associated with patient delays. Age, gender, education level, employment status, average household income, insurance status, comorbidities were taken from the previous manuscript [19], and minutes to nearest CHC was included as a variable as the association between patient delay and long distances from a healthcare facility has been proven in multiple systematic reviews summarising the risk factors of delays in TB [22–25]. An additional two variables were added that might have confounded the relationship between COVID-19 and TB: the symptoms of cough and fever prompting the initial visit to a healthcare provider. We hypothesized that having a fever or a cough can be associated with delays, as these the presence of these symptoms can prompt an immediate visit to a primary health care provider [26] or can be treated as non-severe symptoms and increase delays/number of encounters to informal providers [23, 24, 27, 28]. Sensitivity analyses to cross-validate findings from the quantile regression were performed by using logistic regression for the same models and are in the supporting information. A cut-off of 30 days was used to indicate patient delay (Table A in S1 Text), and a cut-off of 6 encounters was used to indicate a high number of encounters (Table B in S1 Text), with all the same predictors as mentioned above.

### Ethical review

Ethical clearance for this manuscript study was provided by the McGill University Faculty of Medicine and Health Sciences Institutional Review Board (IRB Internal Study Number: A04-M43-22A), Research Institute of McGill University Health Centre (Covid BMGF / 2021–7197), and Universitas Padjadjaran Research Ethics Committee (166/UN6.KEP/EC/2021). Ethics and scientific approval by the institution at the local study site had already been obtained at the time of analysis.

## Results

### Baseline characteristics

The baseline characteristics of the study participants is presented in Table 1. Finally, 149 observations are included from the COVET study and 225 individuals are included from the INSTEP project. The median age of the participants was 35 years and 36 years, before and during COVID-19, respectively. Additionally, 124 (55.1%) participants from the pre-COVID-19 sample were male, whereas 79 (53%) participants from the during COVID-19 sample were male. A hundred and forty-four participants (64.0%) had completed high school in the pre-COVID-19 sample while 72 participants (48.0%) had completed high school in the during COVID-19 sample. A lower number of participants, 64 (28.4%) were unemployed in the pre-COVID-19 sample, whereas 71 (47.7%) participants were unemployed in the during COVID-19 sample. Median monthly household income was lower for the pre-COVID-19 sample as compared to the during-COVID-19 sample (163USD vs 193USD). Less participants were enrolled in an insurance scheme (either the national insurance scheme or a private insurance scheme) pre-COVID-19 as compared to during COVID-19 (74.7% vs 85.9%). The proportion of participants with 1 or more comorbidities was 17% pre-COVID-19 and 21% during COVID-19. All participants from the during COVID-19 sample suffered from symptoms that prompted them to visit a healthcare provider to get a diagnosis for their illness, and there were 9 participants (4%) in the pre-COVID-19 sample that were able to get a diagnosis from a referral without suffering any symptoms.

**Table 1:** Baseline characteristics. Pre-COVID-19 sample and during COVID-19 sample.

	Pre-COVID-19 (Years: 2017–2019) n (%)	During COVID-19 (Years: 2021–2022) n (%)	p-value
	(N = 225)	(N = 149)	
**Age at treatment initiation**			
Median (IQR)	35.0 (24.0 to 50.0)	36.0 (25.0 to 58.0)	0.159
**Sex**			
Male	124 (55.1)	79 (53.0)	0.771
Female	101 (44.9)	70 (47.0)	
**Highest Education Level**			
No formal schooling/less than primary school	52 (23.1)	55 (36.9)	0.007
High school completed	144 (64.0)	72 (48.3)	
College/university completed	29 (12.9)	22 (14.8)	
**Employment Status**			0.001
Unemployed	64 (28.4)	71 (47.7)	
Employed	108 (48.0)	42 (28.2)	
Student at school/university	11 (4.9)	15 (10.1)	
Other (housewife/husband, retired, etc.)	42 (18.7)	21 (14.1)	
Avg. monthly household income (USD)*			
Median (IQR)	163.3 (100.0 to 214.2)	193.3 (102.7 to 333.3)	0.049
**Enrolled in an insurance scheme (private or government)**			
No insurance	57 (25.3)	21 (14.1)	0.013
Has insurance	168 (74.7)	128 (85.9)	
**Smoked in the past year**			0.567
No	131 (58.2)	92 (61.7)	
Yes	94 (41.8)	57 (38.3)	
**Any comorbidities**			
No comorbidities	187 (83.1)	117 (78.5)	0.328
1 or more comorbidities	38 (16.9)	32 (21.5)	
**Symptoms that prompted visit to healthcare provider**			
Cough	188 (83.6)	138 (92.6)	0.016
Night sweats	124 (55.1)	75 (50.3)	0.424
Coughing Blood	47 (20.9)	35 (23.5)	0.640
Weight Loss	148 (65.8)	96 (64.4)	0.875
Fever	139 (61.8)	71 (47.7)	0.01
**Symptom Status**			
No symptoms	9 (4.0)	0 (0.0)	0.033
Symptoms present	216 (96.0)	149 (100.0)	
**Sector of provider at first encounter**			
Private Sector	197 (87.6)	141 (94.6)	0.005
Public Sector	28 (12.4)	8 (5.4)	
**Provider at first encounter**			
Informal Provider	88 (39.1)	72 (48.3)	0.137
Community Health Centre	26 (11.6)	8 (5.4)	
Private Practitioner	81 (36.0)	52 (34.9)	
Private Hospital	28 (12.4)	17 (11.4)	
Public Hospital	2 (0.9)	0 (0.0)	
**Location of diagnosis**			
Community Health Centre	0 (0.0)	1 (0.7)	0.005
Private Practitioner	120 (53.3)	61 (40.9)	
Public Hospital	7 (3.1)	0 (0.0)	
Private Hospital	98 (43.6)	87 (58.4)	
**Location of treatment provision**			
Community Health Centre	55 (24.4)	54 (36.2)	0.001
Private Practitioner	66 (29.3)	3 (2.0)	
Public Hospital	0 (0.0)	2 (1.3)	
Private Hospital	104 (46.2)	90 (60.4)	
**Diagnosis given in the same location as the first encounter**			
Yes	72 (32.0)	63 (42.3)	0.055
No	153 (68.0)	86 (57.7)	
**Treatment given in the same location as the site of diagnosis**			
Yes	149 (66.2)	88 (59.1)	0.194
No	76 (33.8)	61 (40.9)	

* Exchange rate of 1 USD = 15 000 IDR [29], rounded for ease of analysis

The providers most often seen at first encounter by both samples were informal providers, namely pharmacies, traditional healers, midwives. A higher proportion of individuals in the during-COVID-19 sample were given a diagnosis at the same place as their first visit (42.3% vs. 32%), but less treatment initiation occurred at the same place as diagnosis in the during-COVID-19 sample (59.1% vs. 66.2%).

### Distribution of delays, pre-COVID-19 and during-COVID-19

**Table 2** shows the distribution of the number of days for each type of delay. While it took median 28 days for a participant in the pre-COVID-19 sample with symptoms to visit a healthcare provider, it took median 32 days for a participant in the during COVID-19 sample (**Table 2**). The distributions of doctor delays are quite similar, with the pre-COVID-19 median doctor delay of 15 days, and the median doctor delay during COVID-19 as 18 days. Treatment delay was relatively unchanged, from 1 day (IQR 0–4 days) in the pre-COVID-19 sample to 1 day (IQR 0–3 days).

**Table 2:** Delays throughout the care cascade.

Delay	Time point	Median (days)	IQR	95% CI*	Min, Max	p-value**
**Patient Delay**	**Pre-COVID-19**	28	10 to 31	(16, 30)	0, 304	0.001
	**During-COVID-19**	32	14 to 90	(31, 55)	1, 585	
**Doctor Delay**	**Pre-COVID-19**	15	4 to 41	(12, 22)	0, 362	0.253
	**During-COVID-19**	18	5 to 48	(14, 26)	0, 288	
**Treatment Delay**	**Pre-COVID-19**	1	0 to 4	(1, 2)	0, 40	0.774
	**During-COVID-19**	1	0 to 3	(1, 2)	0, 37	
**Total Delay**	**Pre-COVID-19**	52	2 to 452	(45, 63)	2, 252	0.008
	**During-COVID-19**	75	3 to 591	(54, 94)	3, 591	

* Method = “exact”

** Chi-squared test to test difference of medians between the two time-points

### Care pathway for individuals with TB, pre-COVID-19 and during COVID-19

**Fig 3:**
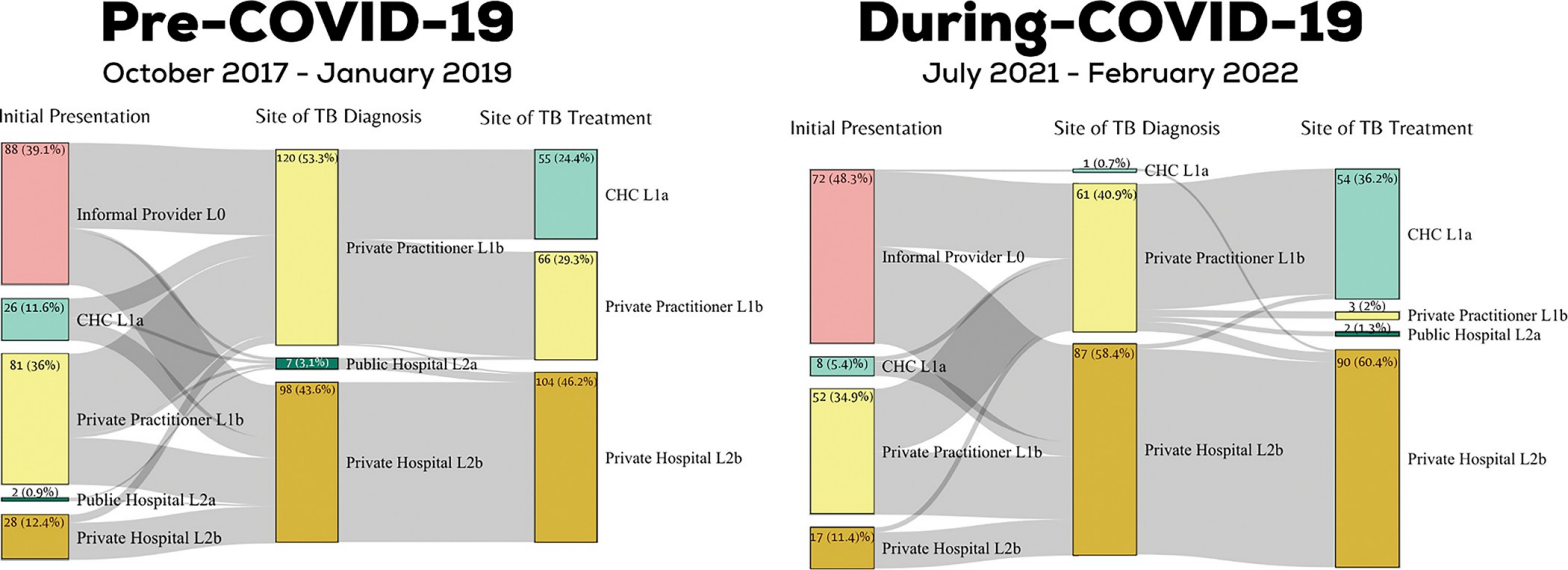
Sankey Chart showing individuals with TB in the pre-COVID-19 and during-COVID-19 sample moving through the care pathway.

For initial presentation, a higher proportion of participants went to informal providers in the during-COVID-19 sample (48.3%) as compared to the pre-COVID-19 sample (39.1%), as depicted in **Fig 3.** The proportion of participants who went to community health centres decreased during COVID-19 (from 11.6% to 5.4%), and no participants went to a public hospital for initial care seeking. More diagnoses happened at private hospitals during COVID-19 (58.4% during-COVID-19 as compared to 43.6% pre-COVID-19) and no diagnoses happened at public hospitals, as compared to 3.1% in the pre-COVID-19 sample. A larger number of individuals with TB got their treatment initiated by CHCs during COVID-19 as compared to before the pandemic. In the pre-COVID-19 sample, out of the 120 individuals diagnosed at private practitioners, 55 (45.8%) individuals were referred to CHCs for treatment management and 65 (55.2%) of individuals stayed with private practitioners. However, in the during-COVID-19 sample, out of 61 individuals diagnosed at private practitioners, 53 (86.9%) went to CHCs or their treatment, 3 (4.9%) stayed with private practitioners. A similarity between the two samples is that most individuals who were diagnosed at private hospitals in the during COVID-19 sample stayed with private hospitals for their treatment management.

### Encounters with health care providers, site of diagnosis provision and missed detection for individuals with TB, pre-COVID-19 and during COVID-19

Median encounters until TB diagnosis was 5 for participants in the pre-COVID-19 sample, and it was 7 in the during-COVID-19 sample. 75% of the sample was diagnosed at encounter 7 in the pre-COVID-19 sample, whereas 75% of the during-COVID-19 sample was diagnosed at encounter 11. Informal providers were also visited until later encounters during COVID-19, and by a higher proportion of participants. There were more missed opportunities to be diagnosed at private practitioners in the pre-COVID-19 sample, but there were more missed opportunities to be diagnosed at private hospitals during COVID-19.

**Fig 4:**
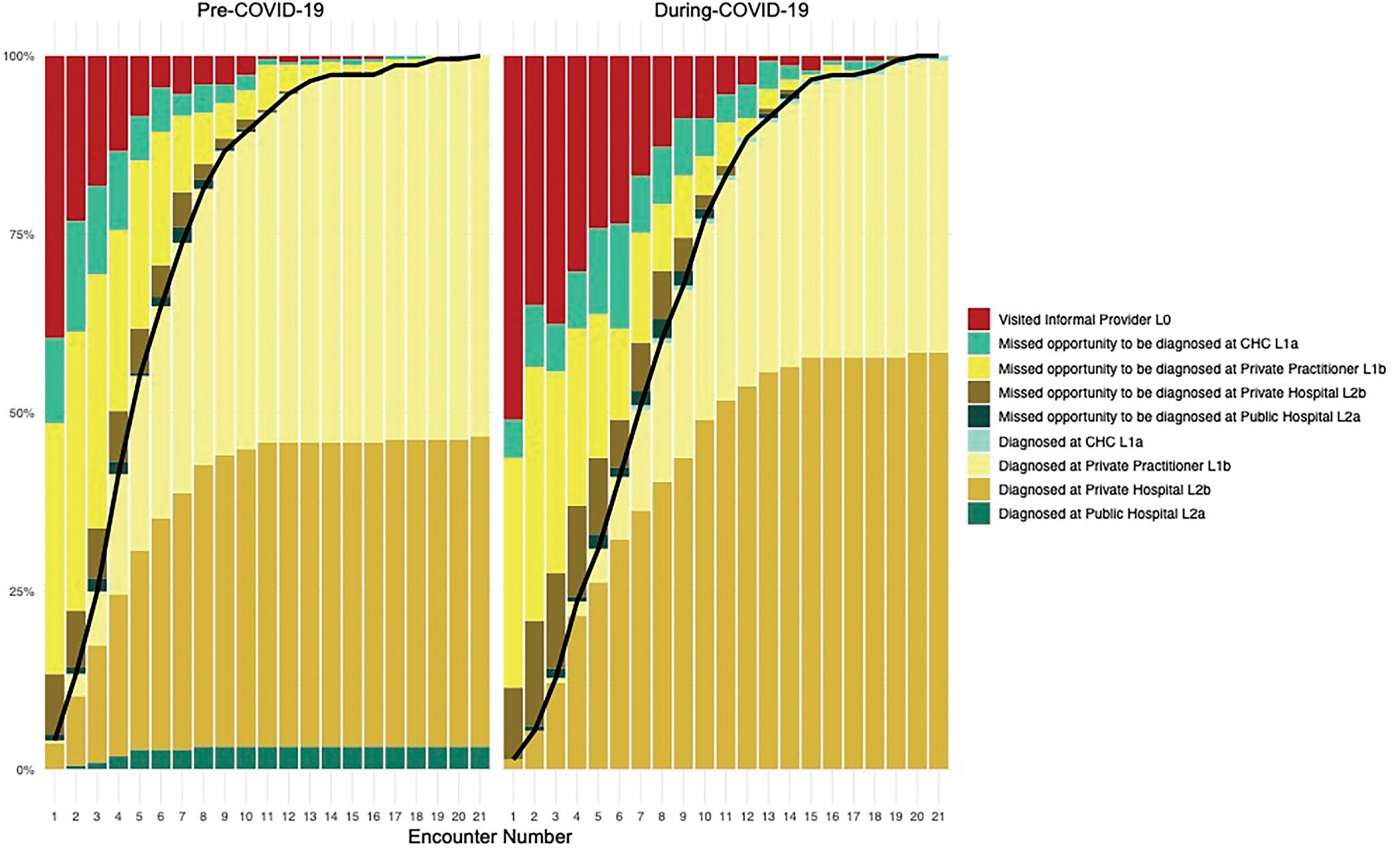
Stacked bar chart of encounters with health care providers and site of diagnosis provision and misses for individuals with TB, pre-COVID-19 sample and during COVID-19 sample.

**Fig 4** shows the proportion of individuals who were either given a diagnosis or missed a diagnosis, over sequential encounters. The black line separates the individuals already diagnosed from the individuals who haven’t been diagnosed yet. This line is steeper for the pre-COVID-19 sample as compared to the during COVID-19 sample, meaning individuals in this sample were diagnosed after a fewer number of encounters as compared to during COVID-19.

### Pathway matrix of individuals with TB, pre-COVID-19 and during COVID-19

**Fig 5:**
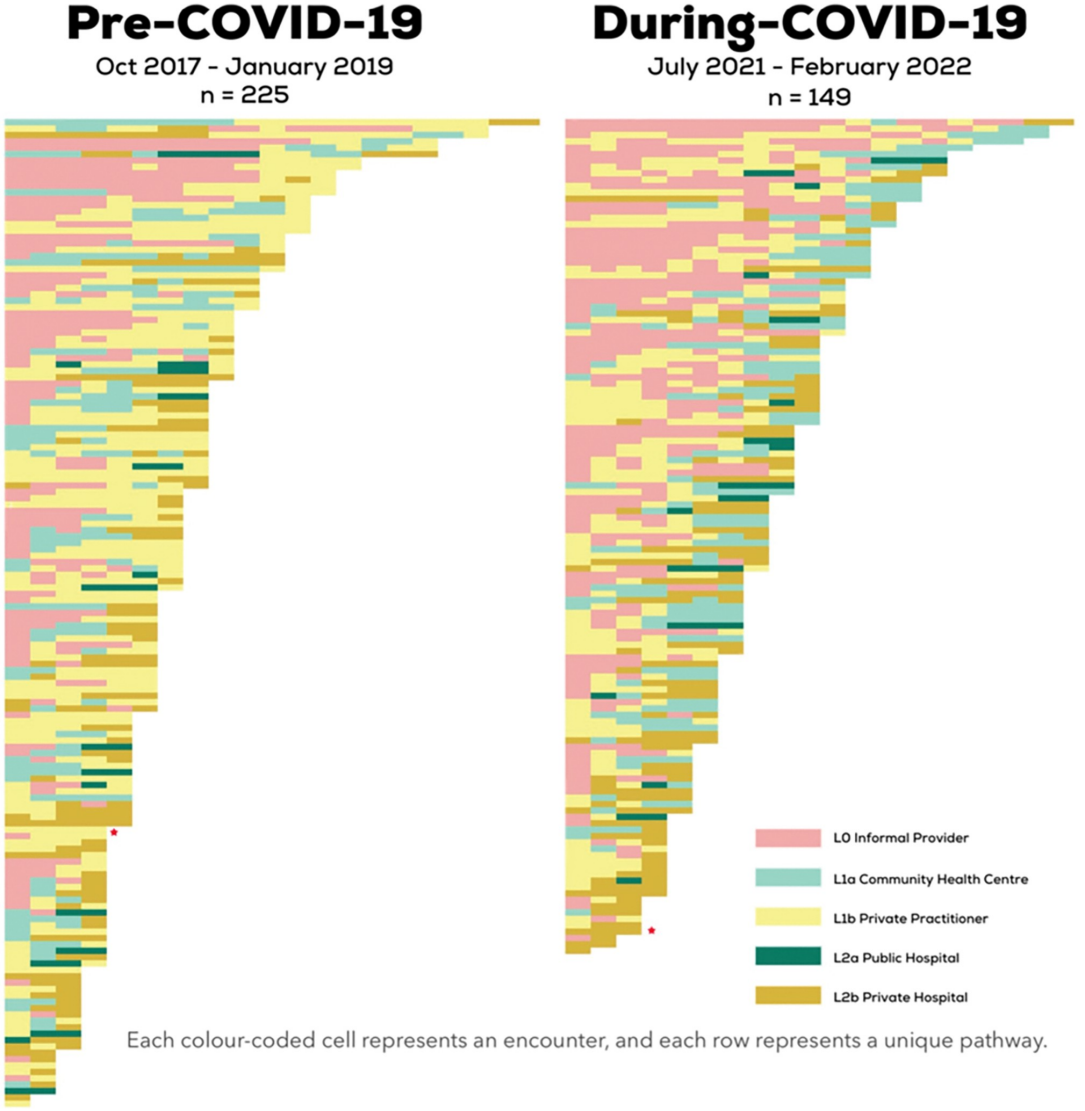
Pathway matrix showing individual encounters with different providers in unique pathways, pre-COVID-19 sample and during COVID-19 sample.

**Fig 5** shows the unique pathways that individuals with TB followed before they were diagnosed with TB. In both matrices, we can see that there are more red cells higher up, indicating visits to informal providers with participants with a higher number of encounters. Visits to informal providers were repeated, during both time points. For participants who had more than 7 encounters, 28% of the visits were with informal providers in the pre-COVID-19 sample, whereas 40% of the visits during COVID-19 were with informal providers. The penultimate visits for the pre-COVID-19 sample took place more often in the private sector, but there were more penultimate visits with CHCs for the during COVID-19 sample. Participants in the post-COVID-19 sample seemed to be switching into the public sector from the private sector more often, as evidenced by the prevalence of green cells near the right-side of the matrix. To quantify this, we calculated the location of the last two encounters of the participants who had more than 7 total encounters. In the pre-COVID-19 sample, 58% of the visits were with private practitioners, whereas during COVID-19, only 7% of these last two visits were with private practitioners; the new mode was CHCs, and 44% of the visits where with CHCs.

### Factors associated with patient delay, doctor delay, and treatment delay

A complete analysis of the median change in patient, doctor, and treatment delay (in days) is presented in **Table 3**. The median change in patient delays in the adjusted model was found to be 2.75 days higher for the during-COVID-19 sample as compared to the pre-COVID-19 sample (adjusted median: 4.42, 95% CI: -7.20, 16.03, p value: 0.457). Individuals who were employed faced lower patient delays compared to individuals who were unemployed (adjusted median: -20.13, 95% CI: -39.14, -1.12, p value: 0.039). Individuals who had greater than six encounters before diagnosis faced a median of 36.68 days higher doctor delays as compared to individuals who had six or less encounters before diagnosis (adjusted median: 36.68, 95% CI: -24.1, 49.25, p value: 0.001). Similarly, individuals with more than six encounters before diagnosis faced higher total delays as compared to individuals who had six or less encounters before diagnosis (adjusted median: 59.40, 95% CI: 35.04, 83.77, p value: 0.001). No factors were found to be associated with treatment delays.

**Table 3:** Factors associated with patient, doctor, and treatment delay (N = 149).

	Patient Delay				Doctor Delay				Treatment Delay				Total Delay			
Variable	Unadjusted (coefficient (CI))	p-value	Adjusted (coefficient (CI))	p-value	Unadjusted (coefficient (CI))	p-value	Adjusted (coefficient (CI))	p-value	Unadjusted (coefficient (CI))	p-value	Adjusted (coefficient (CI))	p-value	Unadjusted (coefficient (CI))	p-value	Adjusted (coefficient (CI))	p-value
**COVID-19 Status**																
Pre COVID-19	Reference		Reference		Reference		Reference		Reference		Reference		Reference		Reference	
During COVID-19	4.00 (-10.91, 18.91)	.60	4.42 (-7.20, 16.03)	.46	3.00 (-4.83, 10.83)	0.45	-3.56 (-16.07, 8.96)	0.58	23.00 (0.85, 45.15)	0.04	-0.28 (-1.70, 1.13)	0.70	23 (2.77, 43.23)	0.03	-14.62 (-39.71, 10.46)	0.28
**Age at Treatment Initiation**	0.03 (-0.10, .15)	.66	-0.21 (-0.59, .17)	.28	0.08 (-0.16, .33)	0.51	-0.16 (-0.62, .29)	0.48	0.29 (-0.44, 1.01)	0.44	-0.01 (-0.05, .03)	0.61	0.36 (-0.24, 0.96)	0.25	-0.57 (-1.31, 0.17)	0.19
**Gender**																
Male	Reference		Reference		Reference		Reference		Reference		Reference		Reference		Reference	
Female	1.00 (-5.55, 7.55)	.77	8.32 (-3.57, 20.20)	.17	5.00 (-2.10, 12.10)	0.17	3.68 (-10.30, 17.66)	0.61	15.00 (-0.39, 30.39)	0.06	0.02 (-1.09, 1.12)	0.98	15 (-3.29, 33.29)	0.11	13.26 (-9.92, 36.4)	0.21
**Highest Education Level Completed**																
Primary School or less	Reference		Reference		Reference		Reference		Reference		Reference		Reference		Reference	
High School Completed	-2.00 (-17.14, 13.14)	.80	-6.54 (-20.50, 7.42)	.36	0.00 (5.18, .00)	1.00	-5.46 (-17.66, 6.74)	0.38	-19.00 (-37.48, -.52)	0.05	-0.65 (-2.04, .74)	0.36	-18 (-35, 59, -0.41)	0.05	-8.56 (-38.02, 20.89)	0.49
College/University Completed	-1.00 (-12.95, 10.95)	.87	-7.18 (-23.46, 9.09)	.39	1.00 (5.92, .17)	0.87	-11.19 (-30.82, 8.44)	0.27	-6.00 (-30.14, 18.14)	0.63	-0.66 (-2.33, 1.00)	0.43	-9 (-31.16, 13.16)	0.43	-21.66 (-55.40, 12.09)	0.17
**Employment Status**																
Unemployed	Reference		Reference		Reference		Reference		Reference		Reference		Reference		Reference	
Employed	-3.00 (-14.19, 8.19)	.60	-20.13 (-39.14, -1.12)*	.04	-9.00 (-17.27, -.73)	0.03	-1.73 (-18.08, 14.62)	0.84	-24.00 (-43.28, -4.72)	0.02	-0.32 (-1.90, 1.25)	0.69	-22 (-39.74, -4.26)	0.02	-27.45 (-59.82, 4.93)	0.07
Student at school/university	-12.00 (-23.46, -.54)	.04	-28.31 (-60.92, 4.29)	.09	-3.00 (-18.54, 12.54)	0.71	-3.01 (-43.47, 37.44)	0.88	-22.00 (-48.59, 4.59)	0.11	0.33 (-1.57, 2.22)	0.74	-24 (-48.83, 0.83)	0.06	-24.13 (-85.96, 37.71)	0.38
Other (housewife/husband, retired)	0.00 (-10.07, 10.07)	1.00	15.78 (-23.55, 55.10)	.43	-7.00 (-19.71, 5.71)	0.28	-2.23 (-33.63, 29.18)	0.89	-16.00 (-42.57, 10.57)	0.24	1.05 (-1.83, 3.93)	0.48	-15 (-41.00, 11)	0.26	22.12 (-28.74, 72.98)	0.43
**Insurance Status**																
Does not have insurance	Reference		Reference		Reference		Reference		Reference		Reference		Reference		Reference	
Has insurance	-1.00 (-5.62, 3.62)	.67	0.82 (-11.27, 12.91)	.90	2.00 (-10.40, 14.40)	0.75	1.44 (-9.19, 12.07)	0.79	-11.00 (-32.42, 10.42)	0.32	0.27 (-1.34, 1.88)	0.75	-11 (-30.08, 8.08)	0.26	-0.40 (-23.04, 22.24)	0.97
**Minutes to nearest CHC**	0.05 (-0.22, .32)	.72	-0.19 (-0.96, .57)	.63	0.32 (-0.39, 1.04)	0.38	-0.07 (-1.12, .97)	0.89	0.50 (-0.67, 1.67)	0.40	-0.03 (-0.08, .02)	0.26	0.25 (-1.05, 1.54)	0.71	0.01 (-2.42, 2.44)	0.99
**Average Monthly Household Income**	0.00 (-0.03, .03)	1.00	0.01 (-0.03, .05)	.67	0.00 (-0.04, .05)	0.93	0.00 (-0.04, .05)	0.87	0.01 (-0.09, .11)	0.84	0.00 (0.00, .00)	0.40	0.03 (-0.07, 0.12)	0.62	0.03 (-0.06, 0.11)	0.60
**Any comorbidities**																
No	Reference		Reference		Reference		Reference		Reference		Reference		Reference		Reference	
Yes, one or more	0.00 (-11.07, 11.07)	1.00	0.80 (-17.14, 18.75)	.93	6.00 (-5.82, 17.82)	0.32	-3.65 (-21.61, 14.32)	0.69	-7.00 (-32.76, 18.76)	0.60	0.69 (-0.93, 2.30)	0.41	1 (-23.60, 25.60)	0.94	3.27 (-31.15, 37.68)	0.85
**Symptom that prompted visit: Cough**																
Cough not present	Reference		Reference		Reference		Reference		Reference		Reference		Reference		Reference	
Cough present	10.00 (-5.03, 25.03)	.19	4.94 (-10.46, 20.34)	.53	7.00 (-1.38, 15.38)	0.10	0.19 (-11.31, 11.70)	0.97	13.00 (-4.29, 30.29)	0.14	-0.36 (-1.87, 1.14)	0.64	19 (3.34, 34.66)	0.02	9.86 (-11.13, 30.885)	0.38
**Symptom that prompted visit: Fever**																
Fever not present	Reference		Reference		Reference		Reference		Reference		Reference		Reference		Reference	
Fever present	0.00 (-3.98, 3.98)	1.00	2.74 (-7.78, 13.27)	.61	9.00 (2.18, 15.82)	0.01	4.18 (-5.59, 13.96)	0.40	8.00 (-6.00, 22.00)	0.26	0.62 (-0.38, 1.62)	0.23	3 (-14.89, 20.89)	0.74	11.97 (-5.65, 29.59)	0.23
**Number of encounters until diagnosis**																
Six or less					Reference		Reference		Reference		Reference		Reference		Reference	
Greater than six					30.00 (24.61, 35.39)	0.00	36.68 (24.10, 49.25)*	0.00	47.00 (32.29, 61.71)	0.00	0.31 (-0.72, 1.34)	0.55	46 (29.70, 62.30)	0.00	59.40 (35.04, 83.77)	0.00
**Provider at first encounter**																
CHC					Reference		Reference		Reference		Reference		Reference		Reference	
Informal Provider					-11.00 (-33.82, 11.82)	0.35	-13.19 (-36.97, 10.60)	0.28	13.00 (-14.97, 40.97)	0.36	-0.08 (-2.21, 2.05)	0.94	11 (-15.52, 37.52)	0.42	-12.44 (-52.63, 27.76)	0.53
Private Practitioner					-9.00 (-32.15, 14.15)	0.45	-2.39 (-25.92, 21.15)	0.84	6.00 (-23.20, 35.20)	0.69	0.06 (-2.11, 2.23)	0.96	4 (-23.97, 31.97)	0.78	5.71 (-35.29, 46.72)	0.79
Private Hospital					-24.00 (-47.03, -.97)	0.04	-7.29 (-32.06, 17.48)	0.57	-17.00 (-51.53, 17.53)	0.34	1.50 (-1.67, 4.66)	0.36	-22 (-54.35, 10.35)	0.18	1.61 (-46.82, 50.04)	0.95
Public Hospital					-12.00 (-38.24, 14.24)	0.37	6.84 (-12.98, 26.65)	0.50					-5 (-48.96, 38.96)	0.82	40.61 (-13.27, 94.51)	0.15
**Provider at diagnosis**																
Private Practitioner									Reference		Reference		Reference		Reference	
Public Hospital									1.00 (-3.40, 5.40)	0.66	-0.42 (-7.88, 7.04)	0.91	-36 (-76, 4.69)	0.08	-0.44 (-71.02, 70.15)	0.99
Private Hospital									-1.00 (-1.81, -.19)	0.02	-0.79 (-1.85, .26)	0.14	-16 (-34.49, 2.49)	0.09	0.12 (-18.74, 18.99)	0.99

CHC: Community Health Center

*: statistically significant.

### Factors associated with number of encounters before diagnosis

Participants in the during COVID-19 sample underwent a median of 2 more encounters before they were diagnosed with TB as compared to the pre-COVID-19 sample (unadjusted median: 2, 95% CI: 0.829, 3.171, p-value 0.001). After adjusting for relevant confounders, participants in the during COVID-19 sample underwent a median of 1.72 more encounters before they were diagnosed with TB as compared to the pre-COVID-19 sample (adjusted median: 1.59, 95% CI: -0.18, 3.36 p-value 0.080). However, for individuals who visited a private hospital as compared to community health centres for their initial visit underwent a smaller number of encounters (adjusted median: -4.29, 95% CI: -6.76, — 1.881, p-value 0.001). Further results on factors associated with number of encounters prior to TB diagnosis is shown in **Table 4**.

**Table 4:** Factors associated with number of encounters before TB diagnosis.

Variable	Unadjusted (coefficient (CI))	p-value	Adjusted (coefficient (CI))	p-value
**COVID-19 Status**				
Pre COVID-19	Reference		Reference	
During COVID-19	2.00 (0.91, 3.09)	0.00	1.59 (-0.18, 3.36)	.08
**Age at Treatment Initiation**	0.00 (-0.03, .03)	1.00	0.02 (-0.03, .07)	.48
**Gender**				
Male	Reference		Reference	
Female	2.00 (0.58, 3.42)	.00	0.08 (-1.44, 1.59)	.92
**Highest Education Level Completed**				
Primary School or less	Reference		Reference	
High School Completed	-2.00 (-3.29, -.71)	.00	0.19 (-1.56, 1.94)	.83
College/University Completed	1.00 (-1.04, 3.04)	.34	1.20 (-0.87, 3.26)	.26
**Employment Status**				
Unemployed	Reference		Reference	
Employed	-1.00 (-2.52, .52)	.20	-1.09 (-2.93, .74)	.24
Student at school/university	-1.00 (-3.08, 1.08)	.35	-0.11 (-5.87, 5.65)	.97
Other (housewife/husband, retired)	-1.00 (-2.71, .71)	.25	0.86 (-2.30, 4.02)	.59
**Insurance Status**				
Doesn’t have insurance	Reference		Reference	
Has insurance	0.00 (-1.29, 1.29)	1.00	0.28 (-1.60, 2.15)	.77
**Minutes to nearest CHC**	0.00 (-0.06, .06)	1.00	0.03 (-0.07, .13)	.54
**Average Monthly Household Income**	0.00 (0.00, .01)	.26	0.00 (0.00, .01)	.36
**Any comorbidities**				
No	Reference		Reference	
Yes, one or more	0.00 (-1.27, 1.27)	1.00	-0.82 (-2.51, .87)	.34
**Symptom that prompted visit: Cough**				
Cough not present	Reference		Reference	
Cough present	2.00 (0.74, 3.26)	.00	0.68 (-0.90, 2.27)	.40
**Symptom that prompted visit: Fever**				
Fever not present	Reference		Reference	
Fever present	0.00 (-1.16, 1.16)	1.00	0.00 (-1.23, 1.23)	1.00
**Provider at first encounter**				
CHC	Reference		Reference	
Informal Provider	2.00 (0.19, 3.81)	.03	0.68 (-1.56, 2.92)	.55
Private Practitioner	-1.00 (-2.55, .55)	.21	-1.29 (-3.30, .73)	.21
Private Hospital	-3.00 (-4.70, -1.30)	.00	-4.29 (-6.76, -1.81)*	0.00
Public Hospital	-3.00 (-5.91, -.09)	.04	-2.58 (-5.85, .69)	.12

CHC: Community Health Center

*: statistically significant.

## Discussion

This study described the care pathways for individuals with TB in an urban setting in Indonesia before and after the onset of the COVID-19 pandemic. We discovered during the pandemic, individuals prefer seeking care with private (formal or informal) community-level health facilities where diagnostic capacity is limited. The care-seeking of individuals with TB did become more complex during the COVID-19 pandemic as compared to before the pandemic. The care-seeking delays were longer, and on average, more visits to healthcare providers before diagnosis were taking place. The delays and number of encounters were influenced by public health and socioeconomic factors, such as the provider at first encounter and employment status.

The participant demographics were different for the two samples. A greater proportion of participants were unemployed in the during-COVID-19 sample. Unemployment was on the rise nationwide during the pandemic, but particularly affected urban settings in Indonesia, and our sample reflected this change [30, 31]. The health inequities between unemployed and employed individuals have been studied extensively, and being unemployed puts a financial strain on an individual, leading to adverse health effects [32–34]. Poverty exacerbates TB [3, 35, 36], and in our study, we can see the direct impact unemployment has on care-seeking outcomes. Furthermore, while the socio-economic well-being of participants in our study beyond treatment completion is unknown, a mixed-method study from an urban setting in Malawi found that TB-affected households remain vulnerable after treatment completion [37].

The private sector has been the primary sector for TB initial visit since before the pandemic, particularly informal providers such as drug shops or pharmacies [14, 17]. However, it is notable that the proportion of pre-diagnostic visits to informal providers increased during the pandemic, while visits to the public sector providers (CHCs and public hospitals) decreased. Most reasons cited for using the informal sector is convenience, affordability, and social and cultural effects [38]. Furthermore, poor perception towards the public sector have also contributed to this preference [39].^.^In the context of COVID-19, a pharmacy that is closer and more accessible might be even more appealing than publicly administered free clinics when transportation and other non-medical costs are considered. The symptoms similarity between COVID-19 disease and TB may also explain the preference to visit private or informal providers [32, 33]. The perceived stigmatization from having either disease [33, 34], or denial regarding severity of the symptoms could have prevented individuals from visiting a formal healthcare provider.

The increased number of visits to informal providers translated in a higher proportion of missed opportunities to receive early diagnosis and treatment, particularly as these individuals had to transition into either the public sector or higher-level private facilities later on [14, 17]. Treatment is free at DOTs centres, which are mostly at CHCs, which could be the reason for more participants opting for care at CHCs rather than with the private sector they obtained a diagnosis amidst COVID-19. More missed opportunities mean worse health outcomes; the reduction of the overall mortality and morbidity among a population becomes more challenging as care seeking is delayed. Individuals with TB could see poorer prognosis of their disease and could increase transmission with their close contacts [28, 40, 41].

We also observed a slight increase in median patient delay, median number of encounters, and median doctor delay, although doctor and treatment delays during the pandemic were comparable to pre-pandemic situation. The increase of pre-diagnostic delays can be explained by the preference to visit informal providers and the stigmatization mentioned above. On the other hand, our findings of a one-day treatment delay are shorter than pooled estimates of treatment delay found in systematic reviews from several countries [42, 43]. We found that the treatment delay is less compared to pre-diagnostics delay as after diagnosis, most of the patients would have transitioned to the public sector: TB tools and drugs are more accessible in this sector compared to the private sector [17]. A 2020 report showed that only 3.7% of the healthcare facilities that owned GeneXpert are private hospitals [44].

Several factors were identified to have significant associations with TB management delays and multiple encounters. First, patients who first visit private hospitals are four times less likely to have more encounters. This is potentially due to the public sector facilities being preoccupied with COVID-19 management, thus having less capacity to adequately manage non-COVID-19 diseases, including TB [45]. In general, private hospitals also have better diagnostic tools, such as chest radiography and culture. Second, patients who are employed are less likely to experience patient delay. This finding can at least be partially explained by the higher health awareness and income among the employed patients. It has been found in various settings that some individuals were not aware that TB treatment is free of charge, leading to more delays [46–50]. Third, patients who underwent more than six visits to providers are more likely to experience doctor delay, supporting the findings of a previous study in Ghana [50]. Interestingly, after controlling for previously identified associated factors, the COVID-19 pandemic did not have a statistically significant effect on patient, doctor delay, or treatment delay. We observed that this may be related to the overall lower power of the data collected during the pandemic compared to pre-pandemic, as COVID-19 disruptions to the Indonesian health system were reported by several studies [51, 52]. We also could not establish the associations between the delays and previously identified factors, such as age, educational level, and insurance status [50, 53, 54].

There are several implications of our results that can be translated into recommendations for moving forward and mitigating the consequences that arose from the COVID-19 disruptions. Advertising and raising awareness regarding the quick referral from diagnosis to treatment initiation might build faith in the formal health sector and incentivise individuals to stick with their care-seeking journey. A second recommendation would be to meet the individuals where they are and conduct more private sector engagement strategies. If a referral algorithm that connects individuals with presumptive TB from the private sector to diagnostic facilities was strengthened, then delays and number of encounters before diagnosis could decrease, leading to the identification of more TB cases. As most of these patients come to informal providers, their engagement should be prioritized [55]. The placement of point-of-care diagnostic tools and drugs in the formal private healthcare facilities would also be beneficial in reducing the delays, as no transition to the public sector would be needed [17].

This research builds on previously collected data to create unique pre- and during-COVID-19 descriptions on care pathways of individuals with TB. Our study is the first to report on TB care pathways in Indonesia since the onset of the pandemic. Nevertheless, there are several limitations to our study. Recruiting only from private providers is a limitation, as it would have been insightful to also describe the landscape of the public sector. However, to complement previous TB pathway analyses [14, 17, 41], as well as collect information on the dominant health sector in Bandung, we decided to exclusively study those individuals currently being looked after by the private sector. Another limitation of our methodology is that we were unable to conduct an attrition analysis. Differences between non-respondents and participants could have contributed to biased results in either direction. Perhaps individuals who refused to participate had even more complex journeys and therefore did not want to share due to fatigue related to their illness or could have had an unremarkable journey and therefore did not see the need to contribute to the research study. Recall bias was another cause of concern in our study, but we tried to mitigate it by ensuring to recruit participants who had recently been diagnosed with TB (in the past 6 months). Finally, our study takes place in an urban setting in Bandung, Indonesia, meaning that the results might not be representative of all individuals with TB in Indonesia. Furthermore, our study cannot capture the pathways of those individuals who were not diagnosed, or who died before obtaining a diagnosis. On the other hand, while delays to TB care have been extensively researched and studied, there was uncertainty regarding the effect of the pandemic on TB care service delivery and care uptake. Our results enrich our understanding of the impact of the COVID-19 pandemic on the functioning of private healthcare markets and studying care-seeking for TB also informs subjects outside of TB. Recommendations from our study can also advise stakeholders on optimizing public-private mix, as the study identified several types of patient-preferred private facilities that should be prioritized in private sector engagement. Another strength is that we were able to study multiple outcomes using one comprehensive survey tool.

## Conclusion

Our study found that the TB patient pathway during the COVID-19 pandemic became more complex, although the delays and encounters were only slightly increased. Our study could not establish the association between these findings to the pandemic. Instead, several public health and socioeconomic factors were attributed to the increased delays and encounters. Identifying the issues in the TB care pathways, assessing the effect of the COVID-19 pandemic on these care pathways, and providing recommendations on addressing the gaps in care will aid in determining how to deliver people-centered care. Further research is needed to map the implications of the pandemic more comprehensively to TB management in Indonesia.

## Supporting information

S1 Text Sensitivity analysis. (DOCX)

S1 Fig COVID-19 restrictions. (TIFF)
