# Supplementary material for: Care pathways of individuals with tuberculosis before and during the COVID-19 pandemic in Bandung, Indonesia
Source: PLOS Glob Public Health. 2024 Jan 2;4(1):e0002251. doi: 10.1371/journal.pgph.0002251 (PMC10760687; doi:10.1371/journal.pgph.0002251)
Supplement: S1 Text — (DOCX) [file pgph.0002251.s001.docx]

# Supporting information

## S1 Appendix. Sensitivity Analysis

The logistic regression was in concordance with the non-parametric model and shows that regarding patient delays (with the cut-off being 30 days), employment status is a significant predictor of a decrease in delays (adjusted odds ratio: 0.29, 95% CI: 0.12, 0.71, p-value 0.008), meaning an individual who is employed is 71% less likely to be delayed more than 30 days in seeking care for their TB symptoms as compared to an individual who is unemployed, controlling for all the aforementioned variables. Being a student is also protective against being delayed in seeking care for symptoms (adjusted odds ratio: 0.13, 95% CI: 0.02, 0.71, p-value 0.025), as a student is 87% less likely to be delayed more than 30 days.

For the outcome of number of encounters before diagnosis, a cut-off of 6 encounters was chosen. The logistic regression found that the odds of having more than 6 encounters was 2.97 times higher for the during COVID-19 sample as compared to the pre-COVID-19 sample (adjusted odds ratio: 2.97, 95% CI: 1.16, 7.92, p-value 0.025). Being a female participant as compared to a male participant also meant that the odds of having more than 6 encounters was 2.95 times higher (adjusted odds ratio: 2.95, 95% CI: 1.42, 6.32, p-value 0.004). Visiting a private hospital for the initial consultation was seen to be protective (adjusted odds ratio: 0.03, 95% CI: 0.00, 0.18, p-value 0.001).

**Table A. Logistic regression: factors associated with patient delay, cut-off 30 days**

|  | Unadjusted | | Adjusted | |
| --- | --- | --- | --- | --- |
| Outcome: Patient Delay greater than 30 days | | | | |
| Variable | Coefficient (CI) | p-value | Coefficient (CI) | p-value |
| COVID-19 Status |  |  |  |  |
| Pre COVID-19 |  |  |  |  |
| During COVID-19 | 2.22 (1.46, 3.41) | 0.001 | 1.55 (0.67, 3.57) | 0.297 |
| Age at Treatment Initiation | 1.01 (1.00, 1.02) | 0.234 | 0.98 (0.95, 1.01) | 0.162 |
| Gender |  |  |  |  |
| Male |  |  |  |  |
| Female | 1.42 (0.95, 2.14 | 0.090 | 1.51 (0.78, 2.97) | 0.226 |
| Highest Education Level Completed |  |  |  |  |
| Primary School or less |  |  |  |  |
| High School Completed | 0.47 (0.29, 0.75) | 0.002 | 0.65 (0.29, 1.47) | 0.304 |
| College/University Completed | 0.53 (0.27, 1.04) | 0.066 | 0.57 (0.20, 1.59) | 0.285 |
| Employment Status |  |  |  |  |
| Unemployed |  |  |  |  |
| Employed | 0.45 (0.28, 0.72) | 0.001 | 0.29 (0.12, 0.71) | 0.008 |
| Student at school/university | 0.39 (0.15, 0.91) | 0.034 | 0.13 (0.02, 0.71) | 0.025 |
| Other (housewife/husband, retired) | 0.91 (0.50, 1.68) | 0.769 | 2.21 (0.43, 17.07) | 0.38 |
| Insurance Status |  |  |  |  |
| Doesn't have insurance |  |  |  |  |
| Has insurance | 0.74 (0.45, 1.22) | 0.235 | 0.61 (0.28, 1.33) | 0.215 |
| Minutes to Nearest CHC | 1.01 (0.98, 1.04) | 0.552 | 0.96 (0.90, 1.01) | 0.102 |
| Average Monthly Household Income | 1.00 (1.00, 1.00) | 0.688 |  |  |
| Any comorbidities |  |  |  |  |
| No |  |  |  |  |
| Yes, 1 or more | 0.84 (0.49, 1.41) | 0.507 | 0.72 (0.25, 2.03) | 0.537 |
| Symptom that prompted visit: Cough |  |  |  |  |
| Cough not present |  |  |  |  |
| Cough present | 1.33 (0.73, 2.49) | 0.359 | 1.10 (0.44, 2.83) | 0.836 |
| Symptom that prompted visit: Fever |  |  |  |  |
| Fever not present |  |  |  |  |
| Fever present | 1.07 (0.71, 1.61) | 0.756 | 1.45 (0.75, 2.82) | 0.269 |

**Table B. Logistic regression: factors associated number of encounters before diagnosis, cut off 6 encounters**

|  | Unadjusted | | Adjusted | |
| --- | --- | --- | --- | --- |
| Outcome: More than 6 encounters | | | | |
| Variable | Coefficient (CI) | p-value | Coefficient (CI) | p-value |
| COVID-19 Status |  |  |  |  |
| Pre COVID-19 |  |  |  |  |
| During COVID-19 | 2.67 (1.75, 4.10) | 0.001 | 2.97 (1.16, 7.92) | 0.025 |
| Age at Treatment Initiation | 1.01 (0.99, 1.02) | 0.335 | 1.01 (0.98, 1.04) | 0.591 |
| Gender |  |  |  |  |
| Male |  |  |  |  |
| Female | 1.66 (1.10, 2.52) | 0.015 | 2.95 (1.42, 6.32) | 0.004 |
| Highest Education Level Completed |  |  |  |  |
| Primary School or less |  |  |  |  |
| High School Completed | 0.65 (0.41, 1.04) | 0.070 | 0.65 (0.27, 1.58) | 0.347 |
| College/University Completed | 1.10 (0.57, 2.16) | 0.771 | 1.05 (0.34, 3.22) | 0.936 |
| Employment Status |  |  |  |  |
| Unemployed |  |  |  |  |
| Employed | 0.69 (0.43, 1.11) | 0.127 | 0.74 (0.28, 2.00) | 0.555 |
| Student at school/university | 0.72 (0.30, 1.68) | 0.453 | 0.72 (0.11, 4.52) | 0.716 |
| Other (housewife/husband, retired) | 0.69 (0.38, 1.26) | 0.233 | 0.98 (0.16, 7.94) | 0.983 |
| Insurance Status |  |  |  |  |
| Doesn't have insurance |  |  |  |  |
| Has insurance | 1.21 (0.73, 2.01) | 0.469 | 0.89 (0.37, 2.15) | 0.797 |
| Minutes to Nearest CHC | 0.99 (0.96, 1.02) | 0.504 | 1.03 (0.98, 1.09) | 0.301 |
| Average Monthly Household Income | 1.00 (1.00, 1.00) | 0.459 | 1.00 (1.00, 1.00) | 0.614 |
| Any comorbidities |  |  |  |  |
| No |  |  |  |  |
| Yes, 1 or more | 0.91 (0.54, 1.54) | 0.738 | 0.95 (0.29, 3.13) | 0.935 |
| Symptom that prompted visit: Cough |  |  |  |  |
| Cough not present |  |  |  |  |
| Cough present | 2.72 (1.40, 5.62) | 0.004 | 1.26 (0.43, 3.84) | 0.671 |
| Symptom that prompted visit: Fever |  |  |  |  |
| Fever not present |  |  |  |  |
| Fever present | 1.10 (0.73, 1.67) | 0.640 | 0.84 (0.40, 1.77) | 0.651 |
| Provider at first encounter |  |  |  |  |
| Community Health Center |  |  |  |  |
| Informal Provider | 1.83 (0.87, 3.88) | 0.113 | 1.05 (0.30, 3.54) | 0.940 |
| Private Practitioner | 0.64 (0.30, 1.37) | 0.242 | 0.36 (0.11, 1.18) | 0.097 |
| Private Hospital | 0.11 (0.03, 0.35) | 0.001 | 0.03 (0.00, 0.18) | 0.001 |
| Public Hospital* | NA | NA | NA | NA |

* Not enough observations for model fitting
