# Supplementary figures and images for: Care pathways of individuals with tuberculosis before and during the COVID-19 pandemic in Bandung, Indonesia
Source: PLOS Glob Public Health. 2024 Jan 2;4(1):e0002251. doi: 10.1371/journal.pgph.0002251 (PMC10760687; doi:10.1371/journal.pgph.0002251)

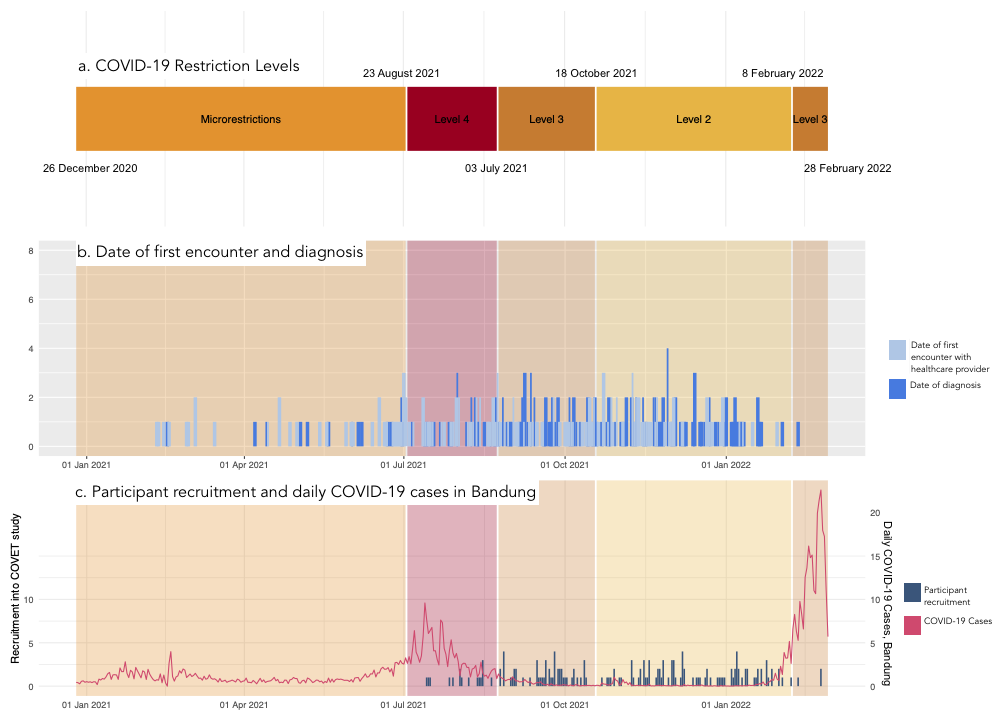

Supplement: S1 Fig — (TIFF) [file pgph.0002251.s002.tiff]
